# Adenosine Receptor Agonists Exhibit Anti-Platelet Effects and the Potential to Overcome Resistance to P2Y_12_ Receptor Antagonists

**DOI:** 10.3390/molecules25010130

**Published:** 2019-12-28

**Authors:** Nina Wolska, Magdalena Boncler, Dawid Polak, Joanna Wzorek, Tomasz Przygodzki, Magdalena Gapinska, Cezary Watala, Marcin Rozalski

**Affiliations:** 1 Department of Haemostasis and Haemostatic Disorders, Chair of Biomedical Sciences, Faculty of Health Sciences, Medical University of Lodz, Mazowiecka 6/8, 92-235 Lodz, Poland; n.m.wolska@gmail.com (N.W.); magdalena.boncler@umed.lodz.pl (M.B.); dawidpolak1991@gmail.com (D.P.); j.wzorek91@gmail.com (J.W.); tomasz.przygodzki@umed.lodz.pl (T.P.); cezary.watala@umed.lodz.pl (C.W.); 2 Laboratory of Microscopic Imaging and Specialized Biological Techniques, Faculty of Biology and Environmental Protection, University of Lodz, Banacha 12/16, 90-237 Lodz, Poland; magdalena.gapinska@biol.uni.lodz.pl

**Keywords:** platelet, adenosine receptor, adenosine receptor agonist, P2Y_12_ antagonist, anti-platelet therapy

## Abstract

Large inter-individual variation in platelet response to endogenous agonists and pharmacological agents, including resistance to antiplatelet therapy, prompts a search for novel platelet inhibitors and development new antithrombotic strategies. The present in vitro study evaluates the beneficial effects of three adenosine receptor (AR) agonists (regadenoson, LUF 5835 and NECA), different in terms of their selectivity for platelet adenosine receptors, when used alone and in combination with P2Y_12_ inhibitors, such as cangrelor or prasugrel metabolite. The anti-platelet effects of AR agonists were evaluated in healthy subjects (in the whole group and after stratification of individuals into high- and low-responders to P2Y_12_ inhibitors), using whole blood techniques, under flow (thrombus formation) and static conditions (study of platelet activation and aggregation). Compared to P2Y_12_ antagonists, AR agonists were much less or not effective under static conditions, but demonstrated similar antiplatelet activity in flow. In most cases, AR agonists significantly enhanced the anti-platelet effect of P2Y_12_ antagonists, despite possessing different selectivity profiles and antiplatelet activities. Importantly, their inhibitory effects in combination with P2Y_12_ antagonists were similar in high- and low-responders to P2Y_12_ inhibitors. In conclusion, a combination of anti-platelet agents acting via the P1 and P2 purinergic receptors represents a promising alternative to existing antithrombotic therapy.

## 1. Introduction

The leading causes of morbidity and mortality in developed countries are cardiovascular disease and stroke, resulting predominantly from arterial thrombosis dependent on blood platelet hyperreactivity. Anti-platelet therapy would appear to be an obvious solution for the treatment and management of such disorders [1,2]. Several therapeutic options are currently available; however, the problem of efficient and safe therapy is complicated by the frequent development of drug resistance stemming from high inter-individual variation among patients caused by genetic and environmental factors [3,4]. Therefore, there is still a demand for novel platelet inhibitors and new therapeutic options.

ADP is one of the key mediators of both physiological haemostasis and thrombosis, being not only a direct agonist of platelets, but also an important factor released from platelet intracellular structures, enhancing the platelet response initially induced by other activators. Platelets have two ADP receptors on their surface: the P2Y_1_ receptor initiates platelet aggregation, while the P2Y_12_ receptor enhances this process, eventually leading to the formation of a clot. Due to this fact, the P2Y_12_ receptor is the main therapeutic target in anti-platelet therapy targeted at the ADP-dependent activation pathway [5]. Generally, the most commonly used clinically approved P2Y_12_ inhibitors include the thienopyridine-class inhibitors (ticlopidine, clopidogrel and prasugrel), the ATP analogue cangrelor, and the cyclo-pentyl-triazolo-pyrimidine derivative ticagrelor [3,5]. Thienopyridines are prodrugs: their short-lived active metabolites irreversibly inactivate the receptor and consequently inhibit ADP-induced platelet activation. Cangrelor is the first (recently approved) intravenous P2Y_12_ receptor inhibitor that reversibly and non-competitively blocks ADP signalling [6].

Adenosine is an important purine metabolite, serving not only as a component of nucleic acids and ATP, the most important energy carrier in the cell, but also as a signalling molecule regulating many cell processes [7,8]. Adenosine receptors (AR) are a subfamily of highly conserved G protein-coupled receptors located in the membranes of various cells and with different physiological functions. Of the four known AR subtypes (A_1_, A_2A_, A_2B_ and A_3_) only A_2A_ and A_2B_ are expressed in platelets [7,9]. Activation of platelet AR results in the enhancement of intracellular cAMP levels and, consequently, the inhibition of platelet activation and aggregation [10].

Apart from the natural agonist adenosine, a group of synthetic, long-lasting agonists were developed [11]; of these, some display good selectivity for A_2A_ or A_2B_ receptors, and others are non-selective compounds activating more than one type of adenosine receptor. One of the oldest known anti-platelet adenosine analogues is 2-chloroadenosine [12]. Other AR agonists were also previously described in the literature as platelet aggregation blocking compounds [12,13,14].

In clinical practice, efficient anti-platelet treatment is often hindered by reduced sensitivity to many anti-platelet agents. To avoid higher drug doses, and therefore a higher risk of bleeding, combined therapy based on the administration of two or more drugs acting on different platelet activation pathways is often used as an alternative. An example of such an approach is the combined administration of acetylsalicylic acid (an inhibitor of thromboxane A2 formation) and clopidogrel (an inhibitor of the P2Y_12_ receptor). Unfortunately, such treatment is still beset by the problem of resistance, especially among patients with type 2 diabetes, i.e., a group at higher risk of thromboembolic events [15,16,17]. We recently proposed a novel approach based on the simultaneous application of two anti-platelet agents, a P2Y_12_ antagonist and an AR agonist, which was found to deepen the action of P2Y_12_ antagonist [18].

The aim of this study is to further explore the potential of combined anti-platelet therapy consisting of simultaneous P2Y_12_ inhibition and adenosine receptor agonization. The lowered dosage of P2Y_12_ inhibitors could potentially reduce side-effects (e.g., excessive bleeding), while the addition of AR agonization would provide adequate anti-platelet effect, and therefore excessive clot formation prevention. We used in vitro methods (whole blood electrical aggregometry, flow cytometry measurement of P-selectin and active form GPIIbIIIa expression, and aggregation/adhesion under flow conditions) to evaluate anti-platelet effects of P2Y_12_ inhibitors: cangrelor and prasugrel (active metabolite R-138727), as well as AR agonists: NECA (non-selective agonist activating both A_2A_ and A_2B_, with strong antiplatelet effect), regadenoson (agonist selective for A_2A_, with moderate antiplatelet effect), and LUF5835 (agonist preferentially activating A_2B_, with a weak antiplatelet effect) in single (either P2Y_12_ antagonist or AR agonist alone) and dual (P2Y_12_ antagonist and AR agonist applied simultaneously) systems.

In the present study, we found that the use of AR agonists can lead to significantly higher inhibition of platelet function caused by P2Y_12_ antagonists and effect was on the same level in high- and low-responders to P2Y_12_ inhibitors, which suggests that AR agonists have the potential to overcome resistance to P2Y_12_ blockers.

## 2. Results

### 2.1. Effects of AR Agonists on Platelet Viability

To ensure that AR agonists used in this work do not exhibit cytotoxic effects on blood platelets, which could influence the results of functional tests, platelet viability was measured. The assay was performed on resting platelets preincubated with NECA, regadenoson and LUF5835. Positive control (1% paraformaldehyde) decreased the fraction of viable cells by 81% on average (*p* < 0.01), while none of the AR agonists exhibited cytotoxic effect (viable cells fraction was not decreased in comparison to the control of non-treated platelets) (Figure S1). This ensures that the anti-platelet effect observed in further research is not due to decreasing fraction of viable platelets (this result should not be interpreted as an assessment of the AR agonists overall toxicity).

### 2.2. Effects of AR Agonists on Platelet Aggregation

**Figure 1:**
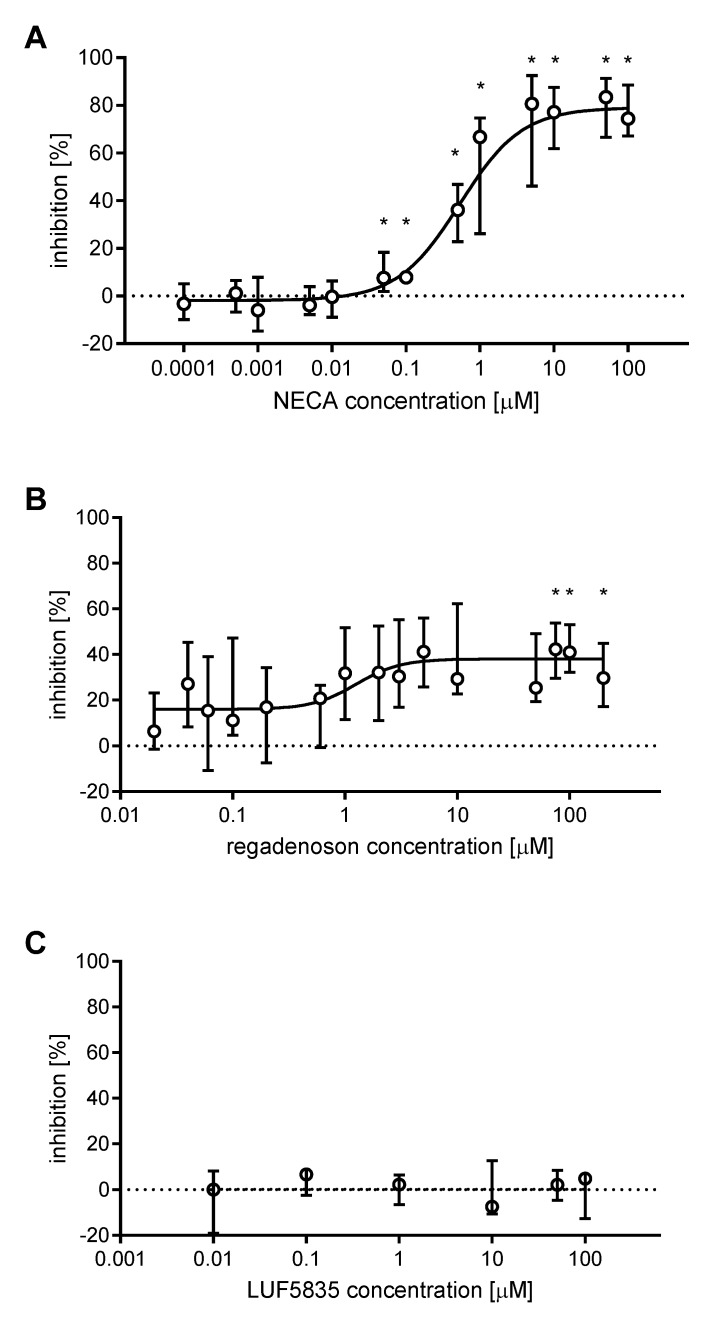
Inhibition of ADP-induced platelet aggregation by AR agonists. Data shown as median ± interquartile ranges, with dose-response plots based on the AUC values using non-linear regression analysis (NECA (**A**) *n* = 5, regadenoson (**B**) *n* = 5; LUF5834 (**C**) *n* = 4). Changes in platelet aggregation were measured in whole blood in response to 10 μM ADP after 3 min preincubation at 37 °C with AR agonist. Data was analysed for statistical significance using repeated measures ANOVA with Geisser-Greenhouse correction and Holm-Sidak’s multiple comparisons test. * indicates statistical significance *p* < 0.05 or lower.

The anti-aggregatory effects of AR agonists NECA, regadenoson, and LUF5835 were evaluated using whole blood stimulated with 10 μM ADP. Dose-response non-linear regression curves were plotted, where possible, to determine the half maximal inhibitory concentration (IC_50_). NECA yielded a curve with the maximal inhibition value of 79.1 ± 4.0%, and IC_50_ of 0.5 µM (95% confidence interval: 0.33 to 0.86) with a coefficient of determination (*R^2^*) equal to 0.876 (Figure 1A). Regadenoson curve had the maximal inhibition value of 38.1 ± 3.2%, IC_50_ of 1.2 µM (95% confidence interval: 0.43 to 3.68), and *R^2^* equal to 0.201 (Figure 1B). LUF5835, unlike the other AR agonists, did not influence platelet aggregation, even at high concentrations - incubation with 50 and 100 μM LUF5835 did not result in a significant inhibition of platelet aggregation (Figure 1C).

### 2.3. Combined Effect of AR Agonists and P2Y_12_ Inhibitors on Platelet Aggregation in Whole Blood

AR agonists were used in a combination with two P2Y_12_ receptor antagonists (one AR agonist + one P2Y_12_ antagonist in each combination): cangrelor and prasugrel metabolite R-138727 (PM). Each compound was used in its IC_50_, with the values taken from our previous work [18]: NECA 0.5 µM, regadenoson 1.2 µM, cangrelor 17 nM, and PM 1.3 µM. In the case of LUF5835, it was not possible to establish an inhibition curve or an IC_50_ value; therefore, a concentration of 100 µM was used.

**Figure 2:**
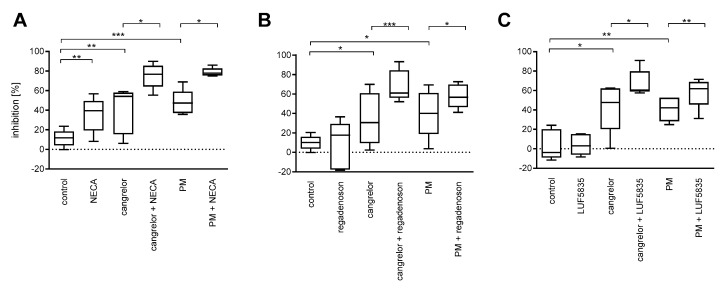
AR agonists intensify the anti-aggregatory effect of P2Y_12_ antagonists: NECA (**A**), regadenoson (**B**), and LUF5835 (**C**). Data are presented as median, interquartile range and minimum and maximum values (*n* = 5 for each AR agonist; totally *n* = 15). Changes in platelet aggregation were measured in whole blood in response to 10 μM ADP after 3 min preincubation at 37 °C with AR agonist and cangrelor, or 15 min preincubation at 37 °C with PM. Statistical significance was estimated by repeated measures ANOVA with Bonferroni’s multiple comparison test, or Friedman’s test with Dunn’s multiple comparison test depending on data distribution. * *p* < 0.05, ** *p* < 0.01, *** *p* < 0.005.

Both P2Y_12_ antagonists significantly reduced platelet aggregation: cangrelor by a mean value of 41% and PM by 46%; however, the percentage inhibition between subjects displayed high coefficients of variation: 54% and 38%, respectively, (*n* = 15). Among the AR agonists, NECA caused a statistically significant decrease in aggregation, whereas regadenoson and LUF5835 did not (Figure 2). Considerable coefficients of variation were also observed (Table S1).

Simultaneous application of an AR agonist was found to intensify the inhibitory effect of P2Y_12_ on platelet aggregation for all (six) combinations; however, the coefficients of variation were also high (Table S1).

### 2.4. Identification of AR Agonist Effect in the Subpopulations of High- and Low-Responders to P2Y_12_ Antagonists

Since platelet sensitivity to P2Y_12_ antagonists was highly differentiated, the numbers of healthy subjects increased to *n* = 20 for each AR agonist and divided into two subpopulations: high-responders and low-responders to P2Y_12_ receptor inhibitors, separately for cangrelor and PM. The cut-off values were medians of the inhibition rates (NECA: 51.6% for cangrelor, 44.7% for PM; regadenoson: 37.9% for cangrelor, 43.5% for PM; LUF5835: 41.9%, for cangrelor, 47.0% for PM). The anti-aggregatory effect of AR agonists on the P2Y_12_ inhibited platelets was then evaluated in the established subpopulations.

**Figure 3:**
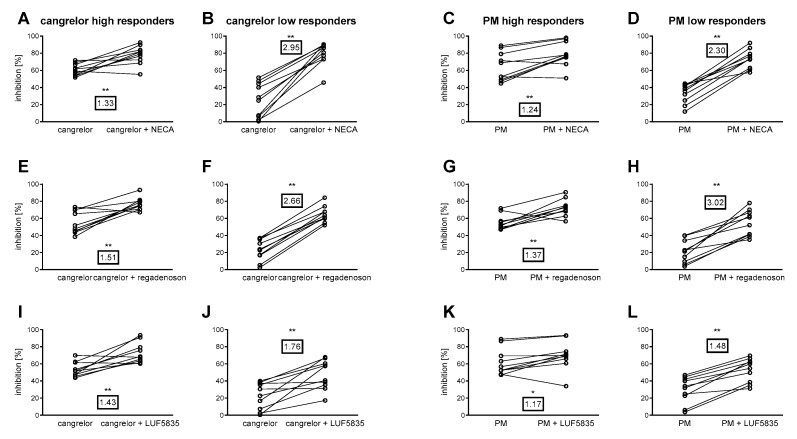
Anti-platelet effect of AR agonists in high- and low-responders to P2Y_12_ antagonists. Combinations of cangrelor (**A**,**B**,**E**,**F**,**I**,**J**), and PM (**C**,**D**,**G**,**H**,**K**,**L**) with NECA (**A**–**D**) regadenoson (**E**–**H**), and LUF5835 (**I**–**L**) are shown. Framed numbers denote the mean inhibition increase factor (arithmetic mean of ratios calculated individually for each donor). Data are shown as pairs of data points (without and with AR agonist) for each blood donor (*n* = 10 in each group). Changes in platelet aggregation were measured in whole blood in response to 10 μM ADP after 3 min preincubation at 37 °C with AR agonist and cangrelor, or 15 min preincubation at 37 °C with AR agonist and PM. Statistical significance between P2Y_12_ inhibitor alone and P2Y_12_ inhibitor with AR agonist groups for each agonist-antagonist pairing estimated by two-tailed Wilcoxon signed rank test. * *p* < 0.05, ** *p* < 0.01.

Significant differences in the inhibition of platelet aggregation were found between the P2Y_12_ antagonist + AR agonist group and the P2Y_12_ antagonist group, for both low- and high-responder groups, for all (six) combinations (Figure 3). The inhibition increase factor, i.e., the number of times an anti-aggregatory effect is intensified by an AR agonist, indicated that the P2Y_12_ antagonist low-responders demonstrated markedly higher inhibition of platelet response caused by AR agonists than the high-responders (Figure 3). Interestingly, combinations of stronger AR agonists (NECA and regadenoson) with P2Y_12_ yielded comparable overall aggregation inhibition in both high- and low-responder groups.

### 2.5. Combined Effect of AR Agonists and P2Y_12_ Inhibitors on Platelet Reactivity

AR agonists were used in combination with two P2Y_12_ receptor antagonists: either cangrelor or prasugrel metabolite R-138727. NECA, regadenoson, cangrelor and PM were used at concentrations equal to previously established IC50 values, while LUF5835 was applied at 100 µM. Both P2Y_12_ antagonists alone significantly reduced platelet activation: cangrelor by a mean value of 53% (P-selectin expression) and by 47% (PAC-1 binding); PM by 42% (P-selectin expression) and by 30% (PAC-1 binding). Observed coefficients of variation in the percentage inhibition by P2Y_12_ antagonists or AR agonists between subjects ranged from 23% to 41% for P-selectin expression and from 14% to 35% for PAC-1 binding (Table S1). Among AR agonists, NECA showed a significant decrease of platelet reactivity (as shown by P-selectin expression), whereas regadenoson and LUF5835 did not significantly affect platelet reactivity in the applied experimental conditions.

**Figure 4:**
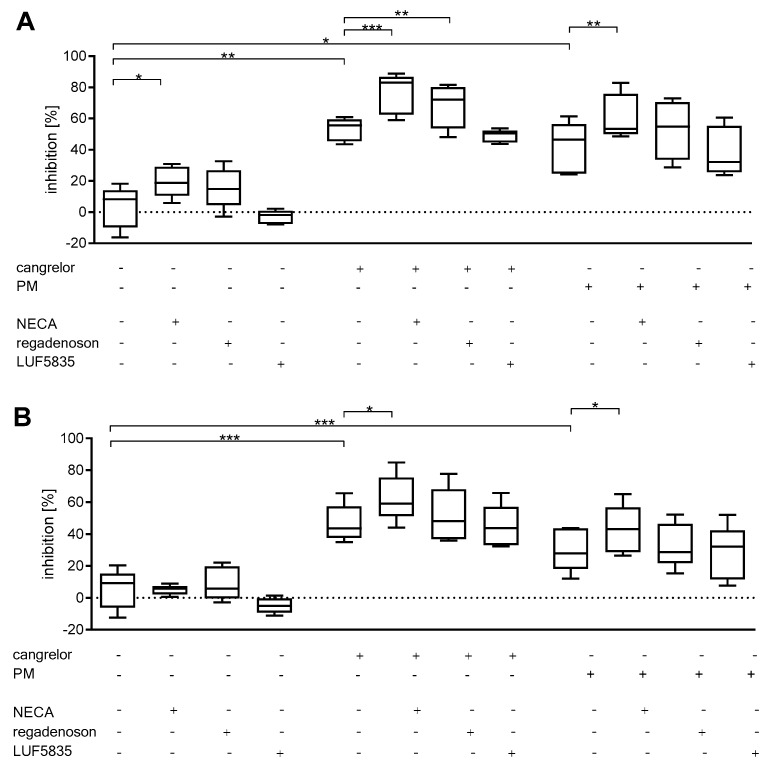
AR agonists deepen the P2Y_12_ inhibitory effect on platelet reactivity as measured by P-selectin expression (**A**) and PAC-1 binding (**B**). Data are given as median, interquartile range, and minimum and maximum values (*n* = 5). Changes in platelet reactivity were measured in whole blood in response to 20 μM ADP after 3 min preincubation at 37 °C with AR agonist and cangrelor, or 15 min preincubation at 37 °C with PM. Statistical significance estimated by repeated measures ANOVA with Bonferroni’s multiple comparison test or Friedman’s test with Dunn’s multiple comparison test depending on the data distribution. * *p* < 0.05, ** *p* < 0.01, *** *p* < 0.005.

The simultaneous application of a P2Y_12_ antagonist and an AR agonist caused significantly greater inhibition of platelet reactivity for cangrelor pairings with NECA and regadenoson (P-selectin only) (Figure 4A)., and PM pairing with NECA (Figure 4A,B). Coefficients of variation were also high in those groups (Table S1 in the Supplementary Material).

### 2.6. Combined Effect of AR Agonists and P2Y_12_ Inhibitors on Thrombus Formation Under Flow Conditions

In the experiments assessing the effects of AR agonists and P2Y_12_ inhibitors on thrombus formation under flow conditions, all compounds were used in concentrations corresponding to the previously established IC_50_ for platelet aggregation, apart from LUF5835, which was applied at 100 µM.

**Figure 5:**
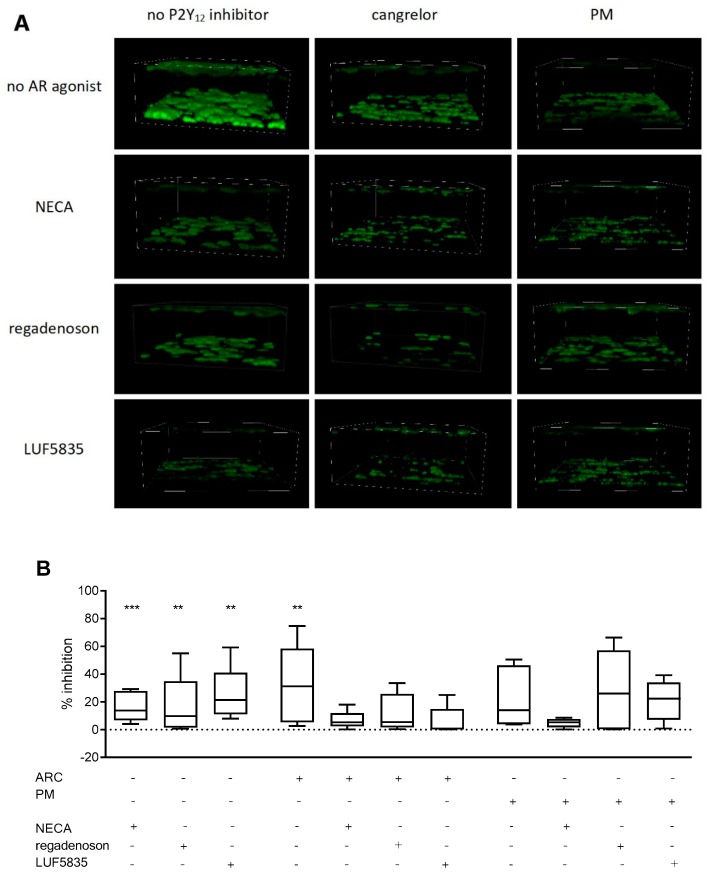
Anti-platelet effect of AR agonists and P2Y_12_ antagonists on thrombus formation under flow conditions. Representative images (**A**) and clot formation inhibition rates (decrease of total clot volume) (**B**). Data are given as median, interquartile range, and minimum and maximum values (*n* = 5). Changes in thrombus formation were analysed in whole blood in response to 60 dyne/cm^2^ flow after 3 min preincubation at 37 °C with an AR agonist and cangrelor, and 15 min preincubation at 37 °C with PM (*n* = 5). The samples were imaged by confocal microscopy and analysed using FIJI image analysis software to obtain summarized clot volumes (for details see Materials and methods). Statistical significance of differences between summarized clot volumes for various AR agonists, P2Y_12_ antagonists and combinations thereof was estimated by the bootstrap-boosted unpaired Student’s t-test. ** *p* < 0.01, *** *p* < 0.005.

Under applied conditions, AR agonists alone decreased clot formation by a mean value of NECA 83.5 ± 10.3%, regadenoson: 83.6 ± 2.0% and LUF5835 74.9 ± 18.9%. P2Y_12_ antagonists alone decreased clot formation by cangrelor (by 68%) and PM (by 77%); however, the obtained variance coefficients for P2Y_12_ inhibitors were high: 103% and 104% respectively. PM treatment did achieve significant inhibition (Figure 5). The mean clot formation decrease for AR agonists was found to be 16.5 ± 11.0% for NECA, 16.4 ± 22.3% for regadenoson, and 25.1 ± 20.0% for LUF5835, all with high CVs (Table S1).

Pairing AR agonists with P2Y_12_ inhibitors did not significantly improve the anti-platelet effect. It should be noted that observed coefficients of variation were also very high in those groups: 151% for cangrelor + LUF5835 (Table S1).

## 3. Discussion

Adenosine receptor (AR) ligands, developed in recent decades, represent a group of agents with anti-inflammatory activity, which can be useful in the prevention and treatment of human diseases [19,20,21]. Promising prospects are emerging for the treatment of arrhythmias, cardiac and cerebral ischaemias, neurodegenerative diseases, inflammation, sleep disorders, pain, diabetes, cancer, renal failure and glaucoma [22]. Some investigated AR agonists were also shown to be potent anti-platelet agents [9,23]. Given the substantial inter-individual variability in response to platelet inhibitors observed in the general population, we hypothesized that the agonists of the A_2A_ and A_2B_ adenosine receptors, which are expressed in platelets, might play a supporting role for anti-platelet drugs, in particular in patients demonstrating poor responsiveness to conventional anti-platelet therapy. The potential advantage of this approach would lie in lowered dosage of traditional anti-platelet drugs, thus avoiding bleeding and other side-effects, while maintaining high protection from thromboembolic events. Therefore, the present in vitro study evaluates the beneficial effects of selected adenosine receptor agonists used in combination with newer P2Y_12_ antagonists, which are administered intravenously (cangrelor) or given orally (prasugrel) during the anti-platelet therapy [24]. More precisely, it examines three AR agonists with molecular structures similar to or different from endogenous adenosine and which demonstrate different selectivity profiles toward A_2A_ and A_2B_ adenosine receptors [22].

Our findings demonstrate that the use of combination of a P2Y_12_ antagonist (cangrelor or prasugrel metabolite) and an AR agonist (adenosine-based compounds: regadenoson, NECA; non-adenosine-based compound: LUF5835) leads to a greater inhibition of platelet function than the P2Y_12_ antagonist alone. What is important is the effect was specific, as the examined AR agonists were not cytotoxic for the cells and their action was much more pronounced in individuals with poor response to P2Y_12_ inhibitors. In a certain sense, this study supports and complements our recent in vitro work, where the anti-aggregatory activity of other AR agonists was examined in the absence or presence of the P2Y_12_ antagonists given above [18].

However, while the two studies have similar aims and target populations, their experimental designs are quite different: they employ different AR agonists and use different methods to determine anti-platelet properties. In addition, the present work was extended with further analyses of the influence of P2Y_12_ antagonists on the effects of the AR agonists, i.e., by comparing groups of high- and low-responders to P2Y_12_ inhibitors. To more closely reflect the in vivo environment, the anti-platelet effects of AR agonists were evaluated using whole blood techniques, under flow (thrombus formation) and static conditions (study of platelet activation and aggregation). However, whole blood aggregometry was used to estimate the inhibitory strength of AR agonists and assess the half maximal inhibitory concentration (IC_50_) values of examined compounds; they were also used to evaluate the anti-aggregatory effects of AR agonists in combination with P2Y_12_ antagonists, which were monitored in a whole population of healthy individuals and in subpopulations of high- and low-responders to P2Y_12_ inhibitors. It is important to note that all AR agonists chosen for the study were tested in a dual model with P2Y_12_ inhibitors, irrespective of the degree of anti-aggregatory activity they exhibited in preliminary experiments. However, the concentration of the AR agonist used for testing with the P2Y_12_ antagonist was chosen based on its anti-aggregatory activity: i.e., regardless of the parameter being estimated, the anti-aggregatory compound was applied at a concentration corresponding to its IC_50_ value. In addition, the AR agonist which did not display antiaggregatory properties was analysed further at the highest used concentration: 100 μM. Cangrelor and prasugrel, being strong P2Y_12_ inhibitors, were used at the IC_50_ values, which were previously reported [18].

The anti-aggregatory potency of the tested compounds was examined at the beginning of the study. Apart from NECA, the anti-platelet effects of regadenoson and LUF5835 were not obvious. It is simply because the anti-platelet properties of regadenoson or LUF5835 have not been explored so far. Regadenoson (CVT-3146, Lexiscan) is a synthetic AR agonist currently approved for clinical use as a pharmacologic stress agent for myocardial perfusion imaging (MPI). It demonstrates non-inferiority to adenosine for detecting reversible myocardial perfusion defects, although it is a moderately selective (K_i_ = 290 nM), short acting A_2A_ AR agonist with low affinity for the remaining adenosine receptors [22,25,26]. In addition to its diagnostic application, regadenoson was considered for the treatment of inflammation and sickle cell disease, and was involved in the development of brain tumor-targeted drug delivery systems [27,28,29]. In contrast, little is known about the biological activity of LUF5835, a synthetic, atypical non-ribose compound, although it was found to display interesting selectivity for adenosine receptors. On the one hand, LUF5835 has a strong ability to activate human A_2B_ adenosine receptor (EC_50_ = 10 nM) but, on the other, it can also interact with human A_1_ (K_i_ = 4.4 nM), A_2A_ (K_i_ = 21 nM) and A_3_ (K_i_ = 104 nM) adenosine receptors [30]. Contrary to LUF5835, NECA is a well-described adenosine analogue possessing high vasodilatory and anti-platelet activity. When compared to adenosine, this non-selective AR agonist, exhibiting high affinity at A_1_ (K_i_ = 14 nM), A_2A_ (K_i_ = 20 nM) and A_3_ (K_i_ = 25 nM) adenosine receptors and lower affinity at A_2B_ AR (EC_50_ = 140 nM), was shown to be over 20,000 times more potent as a vasodilator and 5-10 more effective as an inhibitor of platelet aggregation in response to ADP and adrenaline [22,31]. As a result, NECA is often used as a reference compound; in the present study, it was used as a point of comparison with regadenoson and LUF5835 [29,30,32].

As with NECA, but unlike LUF5835, regadenoson caused significant inhibition of platelet aggregation within the concentration range 1‒100 μM. However, at a concentration of 1 µM or higher, NECA was approximately twice as effective as regadenoson in reducing platelet aggregation (Figure S1). Assuming that the anti-platelet potential of AR agonists corresponds with their respective values of binding constants (K_i_) determined for the A_2A_ adenosine receptor (high affinity receptor), which is preferential over the A_2B_ AR (low affinity receptor) for adenosine [33], regadenoson should have exhibited weaker inhibition in the functional tests than NECA or LUF5835, whereas the anti-aggregatory activity of LUF5835 should be comparable to that observed for NECA. However, in contrast, LUF5835 alone was not found to exert any inhibitory effect. One possible explanation for this inconsistency is that the affinity in LUF5835 binding at A_2A_ adenosine receptor was overestimated. This may be possible, as while the selectivity profile of LUF5835 is only given in one report of Beukers et al. [30], many more reports exist on the binding affinities of NECA and regadenoson, all of which indicate that NECA demonstrates higher affinity for the A_2A_ adenosine receptor than regadenoson: the Ki values were calculated to be within the range of 2.2‒60 nM for NECA [34,35,36] and 290 nM‒1.7 µM for regadenoson [37,38,39]. Therefore, it is not surprising that NECA displayed greater inhibition of platelet aggregation than regadenoson (up to 80%; Figure S1), and was comparable to the high affinity A_2A_-selective AR agonist UK 432097 [18]. Interestingly, a recent work by Fuentes et al. on another group of synthetic AR agonists (PSB family) anti-platelet properties also reported a discrepancy between Ki values and anti-platelet potential, concluding that Ki to A_2A_ receptor is not predictive of those effects [32].

In the light of these observations, further experiments were carried out with NECA and regadenoson at their IC_50_ values (determined in aggregation) and LUF5835 at 100 μM. At the indicated concentrations, the inhibitory effect of AR agonists on platelet activation (5%–19% inhibition) or aggregation (4%–35% inhibition) was considerably lower than observed in studies of platelet thrombus formation on collagen, which reported very high reduction of thrombus volume, i.e., by 75%–84%. NECA appeared to be the most effective AR agonist as it significantly diminished ADP-induced platelet activation, expressed by the fraction of CD62P-positive platelets, ADP-stimulated aggregation, and thrombus formation, compared to controls (DMSO). Regadenoson and LUF5835 only exerted significant inhibitory action against thrombus formation. These observations are mostly in agreement with the study of aggregation by shear stress that showed that some adenosine derivatives, including NECA, increased the blood flow and platelet cAMP level, reduced platelet activation and retention of white blood cells; however, the differences in platelet retention between samples and control were not statistically significant [40].

As regards P2Y_12_ antagonists, all examined parameters were strongly decreased as compared to controls; the degree of inhibition of platelet activation, aggregation and thrombus formation by P2Y_12_ inhibitors reached respectively 52%, 46% and 81%; insignificant changes were only observed in one case, i.e., formation of platelet thrombus in the presence of PM. Overall, the results indicate that compared to P2Y_12_ antagonists, AR agonists were much less effective under static conditions (in models with exogenous ADP), but demonstrated similar anti-platelet activity in flow (without ADP).

Although it is possible that AR agonists may be able to inhibit platelet activation and aggregation more effectively at lower agonist concentration, the present study did not explore the agonist dose-response relationship for obvious reasons: our aim was to obtain high and reproducible cell reaction to ADP; the experimental protocol included five compounds, each of which was tested in various combinations, and more importantly, the samples contained from one to three platelet inhibitors (DMSO, P2Y_12_ inhibitor, AR agonist), depending on the mode of treatment. A desirable cell response was obtained after incubation of whole blood with 10 µM ADP in the aggregation experiments and with 20 µM ADP when studying platelet activation. Such concentrations of ADP may appear high, but they are nevertheless used in studies of platelet function, particularly those concerning aggregation [41,42,43,44].

In most cases, the combination of AR agonists with P2Y_12_ inhibitors led to further inhibition of platelet function. A statistically significant decrease was observed in aggregation experiments, where all compounds improved the action of P2Y_12_ antagonists, and in platelet activation, where the platelet inhibition caused by P2Y_12_ antagonists increased by NECA or regadenoson. These observations are consistent with previous findings indicating that the anti-aggregatory effect of P2Y_12_ inhibitors was enhanced by AR agonists such as UK 432097, 2-Cl-adenosine, MRE 0094 or PSB 0777 [18]. In addition, NECA, regadenoson and LUF5835 were able to increase the inhibition of platelet function caused by the anti-thrombotic activity of P2Y_12_ inhibitors (reduction of thrombus volume was up to 75% compared to P2Y_12_ antagonist alone), although the changes did not reach statistical significance. There are at least two reasons why this might be the case: P2Y_12_ antagonists significantly decreased thrombus volume when applied alone; in addition, very high inter-assay variation was observed. Indeed, the CV values for thrombus volume ranged from 62% to 184% and were substantially higher than the CVs obtained in the remaining assays. In comparison, Ranjit et al. report inter-assay CVs varying from 8% to 638% with regard to blood clot parameters measured by thromboelastography in whole blood, demonstrating the high variability between haemostatic parameters [45].

Taken together, our findings provide further support for the potential therapeutic use of AR agonists in dual anti-platelet therapy in combination with P2Y_12_ receptor inhibitors. Adenosine receptor agonists seem to be an attractive alternative to GPIIbIIIa or P2Y_1_ receptor antagonists, which were demonstrated to have favorable effects on platelet function in combined therapy with thienopyridines [46,47].

High degrees of inter-individual variability were previously observed for the platelet response to agonists (particularly ADP) and anti-platelet drugs [48,49,50,51]; this was also observed in the present study. Therefore, the effect of AR agonists on platelet aggregation was evaluated in high- and low-responders to P2Y_12_ inhibitors using aggregometry: a simple method which allows rapid assessment of platelet response.

All three examined AR agonists increased the anti-aggregatory action of P2Y_12_ inhibitors, allowing their effects to be analysed in the established subpopulations. All the agonists also significantly improved the observed anti-aggregatory effects of P2Y_12_ inhibitors in both high- and low-responders, although the inhibition ratio indicated considerably higher inhibition of platelet aggregation by AR agonists among the P2Y_12_ low-responders than the high-responders (Figure 3). Accordingly, no difference was observed between subpopulations with regard to mean platelet inhibition by cangrelor + AR agonist or prasugrel metabolite + AR agonist (with the exception of LUF5835). Hence, patients resistant to a P2Y_12_ inhibitor may benefit from adjunctive therapy with non-selective or A_2A_-selective AR agonists.

In conclusion, the pharmacological response to anti-platelet agents such as P2Y_12_ receptor inhibitors displays high inter-individual variability, and this prompted the search for new anti-platelet strategies. Our findings show that adenosine receptor agonists may significantly enhance the anti-platelet effect of P2Y_12_ antagonists, despite possessing different selectivity profiles and anti-platelet activities. Significantly, the combination of anti-platelet agents acting via the P_1_ and P_2_ purinergic receptors can be equally effective in both high- and low-responders to P2Y_12_ inhibitors and therefore represents a promising alternative to existing anti-thrombotic therapy.

## 4. Materials and Methods

### 4.1. Chemicals

Adenosine receptor agonists were purchased from Sigma (St. Louis, MO, USA) (NECA (CAS № 35920-39-9)), and Cayman (Ann Arbor, MI, USA) (regadenoson (CAS № 313348-27-5)). LUF5835 (2-amino-6-(1H-imidazol-2-ylmethylsulfanyl)-4-(3-hydroxy-phenyl) pyridine-3,5dicarbonitrile) was synthesized at Laboratory of Molecular Virology and Biological Chemistry, Institute of Medical Biology, Polish Academy of Sciences, Lodz, Poland. Cangrelor (AR-C69931MX) was from Cayman Chemical (Ann Arbor, MI, USA). Prasugrel metabolite (R-138727) was obtained from BOC Sciences (Shirley, NY, USA). Calcein AM was obtained from Molecular Probes (Eugene, OR, USA). Antibodies anti-human CD61/PerCP, CD61/PE, CD62/PE, PAC-1/FITC, mouse IgG1/PE isotype control, mouse IgG1/FITC isotype control, Cellfix, buffered sodium citrate was purchased from Becton-Dickinson (San Diego, CA, USA). Phosphate buffered saline pH 7.4 (PBS) was obtained from Corning (New York, NY, USA). Dimethyl sulfoxide (DMSO), adenosine diphosphate (ADP), and bovine serum albumin (BSA) were obtained from Sigma (St. Louis, MO, USA). All other chemicals, unless otherwise stated, were purchased from Avantor Performance Materials Poland S.A. (Gliwice, Poland).

### 4.2. Chemicals Preparation

The stock and working solutions of cangrelor and prasugrel metabolite were prepared in distilled water. The 100 mM stock solutions of NECA, regadenoson and LUF5835 were prepared in DMSO. Stock solutions were then diluted with PBS to working concentrations not exhibiting precipitates, as noted by Boncler et al. [18], and added to the biological material. The dilution factor was chosen to yield the maximal concentration of DMSO 0.1%, thus the final concentration of DMSO in the biological sample never exceeded 0.1% in any of the assays.

### 4.3. Blood Donors

Experiments were approved by the Ethics of Research in Human Experimentation Committee at the Medical University of Lodz, approval number (RNN/43/17/KE). After having received written consent from volunteers, blood was collected from healthy donors (30 men and 62 women; mean age 29.5 ± 8.8 years) into a vacuum tube containing 0.105 mol/L buffered sodium citrate, with a the final citrate: blood ratio of 1:9 *v*/*v*. All individuals stated that they had not taken medications known to influence platelet function for at least two weeks prior to the study.

### 4.4. Platelet Viability Assay

Platelet viability in the presence of AR agonist and P2Y_12_ inhibitors was assessed in resting platelets according to Rywaniak et al. [52]. Samples were preincubated with AR agonists for 3 min at 37 °C. Positive control (assumed to result in a low platelet viability) was blood preincubated in the presence of 1% paraformaldehyde (PFA) for 15 min at 37 °C. Samples were then diluted 10-fold with PBS pH 7.4, labelled with anti-CD61/PE antibodies (15 min, RT) and subsequently stained with 0.1 μM calcein AM (15 min, 37 °C). The percentage of calcein-negative platelets was measured immediately after staining using flow cytometry, gathering 5000 events (CD61/PE-positive objects), using FACSCanto II flow cytometer (Becton-Dickinson, Franklin Lakes, NJ, USA).

### 4.5. Platelet Aggregometry Measured in Whole Blood

The measurements was performed according to the manufacturer’s instructions. Briefly, whole blood was preincubated with an AR agonist and/or P2Y_12_ inhibitors for 3 (AR agonists and cangrelor) or 15 (prasugrel metabolite) minutes at 37 °C, then 300 µL of blood was transferred into the measurement cell and diluted with 300 μL saline (0.9%) and preheated to 37 °C for another 3 min. Then, 10 μM ADP (final concentration) was added and platelet aggregation was recorded continuously for 10 min using a Multiplate analyser (Hoffmann-La Roche, Basel, Switzerland). Area under the curve (AUC) was analysed. All measurements were completed within three hours of blood collection.

### 4.6. Platelet Reactivity Measured by Flow Cytometry

Whole blood was preincubated with an AR agonist and/or a P2Y_12_ inhibitor for 3 (AR agonists and cangrelor) or 15 (prasugrel metabolite) minutes at 37 °C then platelets were activated with 20 µM ADP (final concentration). Subsequently, a sample was diluted 10-fold with PBS, and labelled with anti-CD61/PerCP, anti-CD62P/PE and PAC-1/FITC antibodies (15 min, RT), then fixed with CellFix for 1 h at RT. Directly before measurement, the samples were diluted 1:1 with PBS and the assay was performed, gathering 10,000 events (CD61/PerCP-positive objects), using FACSCanto II flow cytometer (Becton-Dickinson, Franklin Lakes, NJ, USA).

### 4.7. Thrombus Formation Under Flow Conditions

The effect of the AR agonists and P2Y_12_ inhibitors on thrombus formation was assayed with the use of the Venaflux system (Cellix, Dublin, Ireland) according to the protocol based on studies published elsewhere [53] using Vena8 Fluoro+ biochips. The channels were coated with type I collagen (20 µg/mL) overnight at 4 °C and blocked with 0.1% BSA for 1 h at 4 °C. The biochip was mounted on the stage of an inverted AxioVert microscope thermostatically controlled throughout the experiment to maintain a constant temperature of 37 °C (Carl Zeiss, Oberkochen, Germany). Whole blood, supplemented with D-phenylalanyl-prolyl-arginyl chloromethyl ketone (PPACK) as a thrombin inhibitor (final concentration 0.05 mM), was preincubated with an AR agonist and/or a P2Y_12_ inhibitor for three (AR agonists and cangrelor) or 15 (prasugrel metabolite) minutes at 37 °C. Samples were recalcified with CaCl_2_ (final concentration 1 mM) immediately before measurement. The samples were then perfused through the channels of the chip using a shear force of 60 dyne/cm^2^ for 4 min. The thrombi were stained in channels by washing with 10 µg/mL fluorescein dissolved in PBS for two minutes at 5 dyne/cm^2^. Following this, the samples were perfused with CellFix for 4 min at 5 dyne/cm^2^. Such prepared channels were imaged by confocal microscopy.

### 4.8. Imaging and Image Analysis

Confocal imaging of thrombi was performed with a Leica TCS SP8 confocal microscope with LAS X 2.0.2.15022 software (Leica Microsystems, Wetzlar, Germany) using the objective HC PL IR APO 40×/1.10 (water immersion). The 488 nm supercontinuum white light laser (WLL) (12% intensity) was used to excite the fluorescein-stained thrombi. The emission was collected by a photomultiplier tube detector in the range of 492–564 nm. Confocal Z-stack scans were performed at a rate of 400 Hz, zoom 1.0, pinhole 1.0 and line averaging set at 3 to improve image quality. In each field of a view 70 focal planes were acquired (logical size format X/Y/Z 512/512/70) [54]. For the analysis of images, FIJI software was used [55], according to our previously established protocol [56]. Briefly, the thresholding procedure was performed with the use of ‘Auto Local Threshold’ function (Bernsen method) with a radius value set at 5. To quantify the identified objects, the ‘3D Object Counter’ tool was applied, with a threshold set at 255, and a cut-off set at 20 µm^3^ (to exclude the objects too small to qualify them as thrombi). Volumes of separated thrombi were acquired for further analysis, and subsequently summarized to obtain total clot volume per sample.

### 4.9. Statistical Analysis

The results are expressed as median with interquartile range. The Shapiro-Wilk test and Mauchley’s test were used to test the normality of data distribution and sphericity of variances, respectively. Normally distributed data was analysed with the pairwise Student t-test or two-way analysis of variance for repeated measures with the post hoc Bonferroni’s multiple comparisons test or repeated measures ANOVA with Geisser-Greenhouse correction and Holm-Sidak’s corrections for multiple comparisons test. Data departing from normality, variance sphericity and/or variance homogeneity were assessed with the Wilcoxon’s signed ranks test or Friedman’s test with Dunn’s correction for multiple comparisons. Coefficient of variation (CV) was used to compare the variability across the variables. Variables with extremely high values of CV (thrombus volume) were bootstrapped to ensure that the revealed differences between groups were not observed due to pure chance. The statistical analysis was performed using the following software packages: Statistica v.13 (Dell Software, Round Rock, TX, USA), StatsDirect v.2.8.0 (StatsDirect Ltd., Merseyside, UK) and GraphPad Prism v.5. (GraphPad Software, San Diego, CA, USA)

## Supplementary Materials

The following are available online, Table S1: Coefficients of variation obtained in aggregometric, cytometric, and thrombus formation under flow conditions experiments carried out in single (either P2Y_12_ antagonist or AR agonist alone) and dual (P2Y_12_ antagonist and AR agonist applied simultaneously) systems (*n* = 5 per experimental method).

## Abbreviations

ADP	Adenosine Diphosphate
AR	Adenosine Receptor
cAMP	Cyclic Adenosine Monophosphate
NECA	5′-(N-ethylcarboxamido) adenosine
IC_50_	Half maximal inhibitory concentration
