# Supplementary material for: Adenosine Receptor Agonists Exhibit Anti-Platelet Effects and the Potential to Overcome Resistance to P2Y12 Receptor Antagonists
Source: Molecules. 2019 Dec 28;25(1):130. doi: 10.3390/molecules25010130 (PMC6982709; doi:10.3390/molecules25010130)
Supplement: Supplementary file 1 [file molecules-25-00130-s001.pdf]

Supplementary Materials:

Table S1. Coefficients of variation obtained in aggregometric, cytometric, and thrombus formation under flow conditions experiments carried out in single (either $P2Y_{12}$ antagonist or AR agonist alone) and dual ($P2Y_{12}$ antagonist and AR agonist applied simultaneously) systems (n = 5 per experimental method).

CV [%]	NECA	regadenoson	LUF5835	cangrelor	PM	cangrelor +NECA	cangrelor + regadenoson	cangrelor + LUF5835	PM + NECA	PM + regadenoson	PM + LUF5835
aggregation	22	28	21	54	38	50	47	43	14	20	40
cytometry: P-selectin	28	26	23	31	41	48	50	41	45	38	41
cytometry: PAC-1	19	14	19	35	35	53	50	42	44	38	41
thrombus formation under flow conditions	93	184	110	103	104	150	121	151	128	129	97
